# Intravascular embolization versus surgical resection for patients with scalp arteriovenous fistula

**DOI:** 10.1186/s41016-018-0148-1

**Published:** 2019-01-15

**Authors:** Jianfeng Zheng, Zongduo Guo, Xiaodong Zhang, Xiaochuan Sun

**Affiliations:** Department of Neurosurgery, the First Affiliated Hospital of Chongqing Medical University, NO.1 of Youyi Rd, Yuzhong District, Chongqing, China

**Keywords:** Scalp arteriovenous fistula, *Intravascular embolization*, Surgical resection

## Abstract

**Background:**

Scalp arteriovenous fistula (sAVF) is a rare vascular disease; so far, the standard guidelines for the treatment of sAVF are still unclear. Since its complex vascular anatomical structure, surgical management of sAVF remains an enormous challenge.

**Methods:**

Between March 2016 and October 2017, three patients with sAVF admitted to the First Affiliated Hospital of Chongqing Medical University were reviewed, and clinical characteristics, imaging features, and surgical outcomes were analyzed.

**Results:**

Three consecutive patients with sAVF were admitted to our hospital during the study period. Two patients received intravascular embolization and one patient received surgical resection. No procedure-related complications occurred after successfully treatment. During the long-term follow-up period, three patients recovered well and had no recurrence of lesion.

**Conclusions:**

Either intravascular embolization or surgical resection is an effective therapy method for sAVF. Careful identification of angioarchitecture features contributes to successful treatment for the complex sAVF; therefore, it is recommended that neurosurgeons make the best treatment plan based on the location and number of the fistulas, feeding the artery, and draining the vein.

## Background

Scalp arteriovenous fistula (sAVF) is defined as an abnormal connection without a capillary vessel between the high-flow feeding artery and the low-flow draining veins of the scalp [1–6]. The etiology of sAVF may be congenitally caused or posteriori acquired and, in most patients, caused by a variety of head trauma which showed pulsatile scalp mass with vascular murmur [3, 4, 7–10]. Cerebral angiography is the diagnostic gold standard and can show location and number of the fistulas, expanded feeding arteries, as well as obviously tortuous draining vein. Operation is the main way of treatment for this rare vascular disease, including open surgical resection and intravascular embolization. In this paper, we describe three cases of sAVF who were successfully treated including two with intravascular embolization and one surgical resection. Furthermore, we reviewed the literature on sAVF and discussed the etiology, clinical features, and management strategies for this rare vascular disease.

## Methods

The patient database at the First Affiliated Hospital of Chongqing Medical University was reviewed. The clinical characteristics, imaging features, and operative methods of all patients with sAVF were analyzed. The way of operation was decided by a cerebrovascular team which includes the experienced neurosurgeons and neurointerventional doctors, and the location and number of the fistulas, the feeding artery and draining vein, and the clinical condition and therapeutic preference of the patients were also to be considered. Simultaneously, the literature of sAVF in the past 15 years was summarized. The etiology, surgical methods, and outcome of the patients were investigated.

## Results

Between March 2016 and October 2017, three consecutive patients with sAVF were admitted to our hospital. A pulsating scalp mass accompanied by vascular murmur was observed in the three patients by physical examination. Cerebral angiography was performed before operation to identify feeding arteries and draining vein. One patient was treated using surgical resection, and two patients were treated by endovascular embolization. No procedure-related complications occurred after successful treatment. The three patients recovered well without any recurrence during clinical follow-up. In addition, the etiology, blood supply artery, drainage vein, and fistula of 27 cases of sAVF reported in the literature are shown in Table 1. Among them, interventional embolization was performed in 14 cases, surgical resection in 11 cases, and combination of 2 treatments in 2 cases (Table 2).

**Table 1:** Scalp arteriovenous fistulas in different series

Study	No Patients	Etiology(T/I/C)	Feeder	Drainage	Fistula(S/M)	Surgery(E/R/P)
Ni W et al. [1]	3	T, C, C	STA OA, STA OA FA, STA	STV, STV, STV	S, M, M	E, E, E
Cil B et al. [5]	2	I, T	STA OA, STA	STV	S	P, P
Feng Z et al. [20]	1	T	STA ACA	EJV	S	R
Hee J Ki et al. [10]	1	T	STA	STV	S	R
Katsunori A et al. [6]	1	T	STA	STV	S	R
Xue B et al. [2]	1	T	STA SOA OA PAA	STV	M	R
Champeaux et al. [22]	1	I	STA	STV	S	R
Dabus G et al. [4]	3	I, I, I	ECA, OA, STA	SFV, SS, STV	S, S, S	E, E, E
Yang M et al. [16]	3	C, T, C	STA, STA, STA	STV SV, STV, SV SOV	S, S, S	E, R, R
Anna M et al. [23]	1	T	STA	SV	S	E
Youn SW et al. [13]	1	T	OA MMA	STV	S	P
Dalyai RT et al. [3]	1	C	OA	SV EJV	S	E
Dogan S et al. [14]	1	I	STA	STV	S	E
Senoglu M et al. [18]	1	C	ECA	SS	S	P
Miekisiak G et al. [24]	1	T	STA	OV	S	R
Corr PD et al. [21]	1	T	OA	SS TS	S	R
Feng Li et al. [11]	1	T	STA	STV	S	R
Whiteside et al. [19]	1	T	STA	SV	S	E
Kim DM et al. [7]	1	C	STA	SV	S	E
Amlashi SF et al. [12]	1	I	STA	STV	S	R

*T* trauma, *I* iatrogenic, *C* congenital, *STA* superficial temporal artery, *OA* occipital artery, *SOA* supraorbital artery, *SS* sigmoid sinus, *TS* transverse sinuses, *ECA* external carotid artery, *FA* facial artery, *PAA* posterior auricular artery, *MMA* middle meningeal artery, *EJV* external jugular veins, *SFV* superficial frontotemporal vein, *Sv* scalp veins, *SOV* superior ophthalmic veins, *OV* occipital vein, *S* single, *M* multiple, *E* embolization, *R* resection, *P* puncture

**Table 2:** Treatment of scalp arteriovenous fistulas in the last 15 years

	Total cases	Type A	Type B	Type C	Cure	Recurrence	Complication
Intravascular embolization	14	10	2	2	12	2	2
Surgical resection	11	9	1	1	11	0	1
Combined treatment	2	0	2	0	2	0	0

Type A, a single fistula fed by a single proximal feeding artery; type B, a single fistula fed by multiple arterial feeders; type C, multiple fistulas with plexiform feeding arteries and a main dilated draining vein

### Case 1 report

**Fig. 1:**
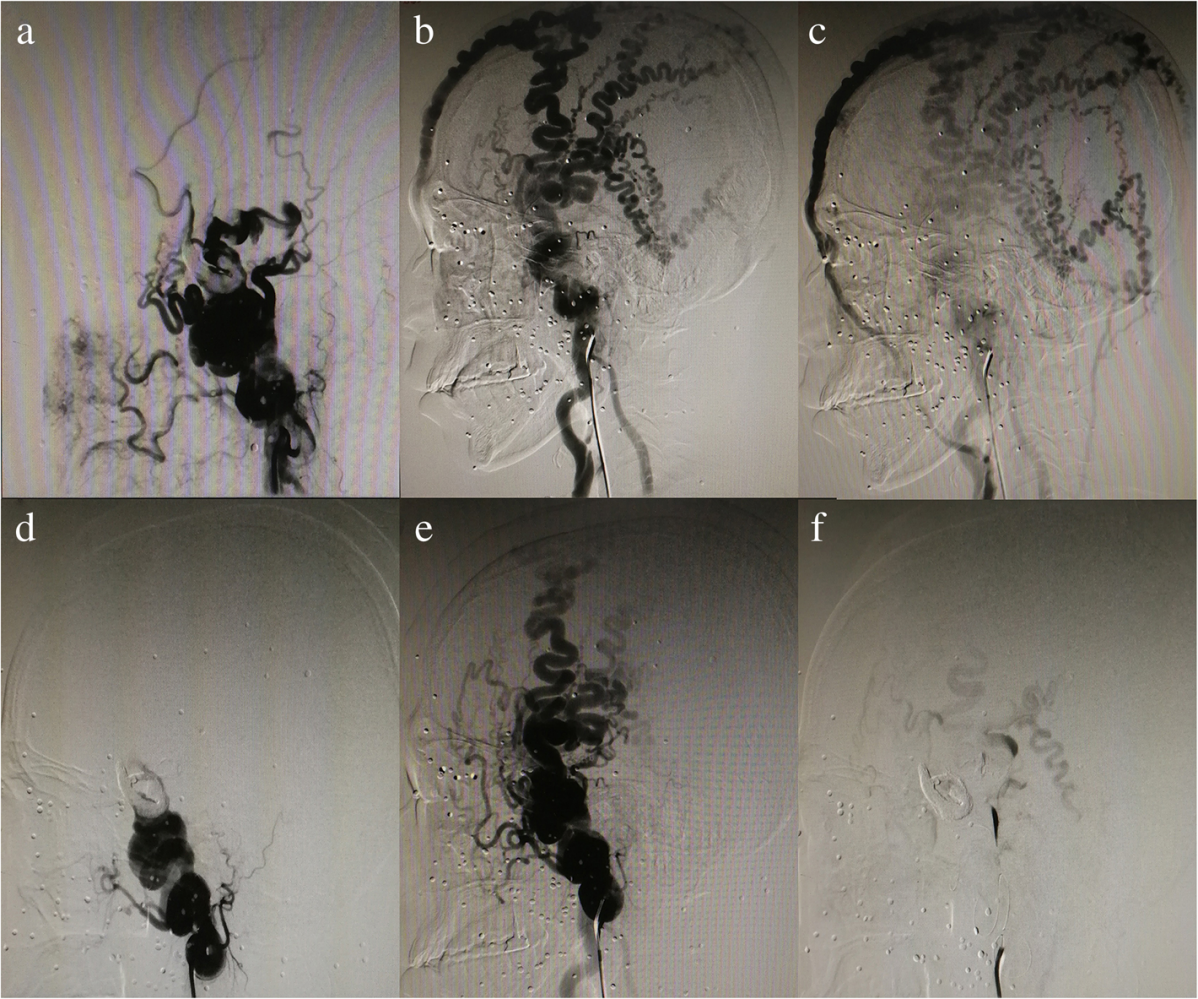
**a**–**c** High-flow sAVF with arterial feeders from bilateral superficial temporal artery and left pulley artery, which drains into an enlarged scalp and facial venous. **d**–**f** Embolization therapy with Onyx-18 liquid material, and the almost occluded fistulous connections

One patient, a 38-year-old man, was admitted to our hospital on March 4, 2016. From 20 years ago, the patient found that a mass in the left frontotemporal part of the head gradually increased in size, which showed fluctuating feeling and blood flow sensation but no any pain or numbness. No other significant medical history but a history of brain trauma from a head gunshot wound 20 years ago. No abnormalities were available on neurologic examination except for blind eyes after head injury. Laboratory examinations, electrocardiogram, chest CT, and abdominal ultrasound also showed no obvious abnormalities. A CT angiography (CTA) was performed immediately after admission, showing abnormal blood vessels in the left frontotemporal scalp. Further digital subtraction angiography (DSA) demonstrated sAVF on this patient (Fig. 1). The patient was optimized and taken up for interventional embolization electively. Detailed informed consent was taken from the patient and his family members. The operation was performed under tracheal intubation and general anesthesia. After a 6F guiding catheter was placed into the left external carotid artery, the headway 17 microcatheter assisted by the synchro-10 microfilaments was used to select the left superficial temporal artery and other branches, and then Onyx-18 liquid agent was used to embolize lesions. Again, angiography showed no signs of AVF in the region of the left frontotemporal scalp and patency of blood flow in the left internal carotid artery. The amount of heparin used in the operation was 45 mg and intraoperative blood loss was less than 10 ml. After operation, all the problems were resolved and no operative complications occurred. The patient was followed-up clinically for nearly 2 years with normal symptoms and signs. Angiography examinations were normal without any recurrence.

### Case 2 report

**Fig. 2:**
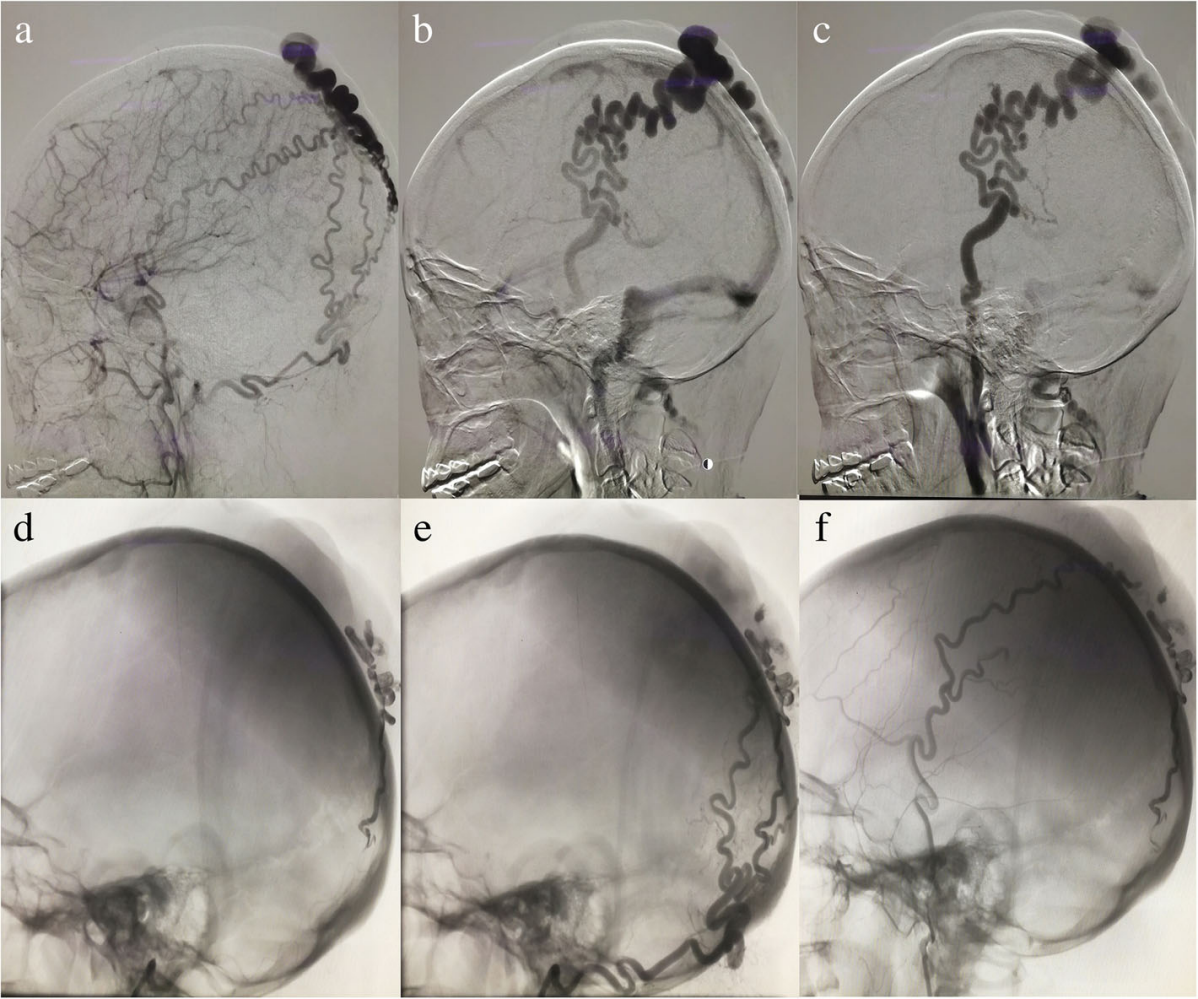
**a**–**c** sAVF having feeders from the markedly dilated bilateral occipital artery and superficial temporal artery, which drain into a tortuous superficial scalp vein. **d**–**f** After Onyx-18 liquid embolic material filling, almost occlusion of the fistulous connections

On July 29, 2016, a 43-year-old female patient was referred to our department due to a pulsating mass in the right top of the head. Twenty-three years ago, the patient found a soft mass with marked vascular fluctuations, but she did not go to the hospital for treatment. One year ago, the mass gradually increased to 4 × 6 cm without swelling, pain, numbness, or bleeding. The patient underwent head CTA, which showed that a serpiginous subcutaneous mass was in the region of the top head scalp. The patient received routine auxiliary examination after entering our department; all test results were within normal limits. Further DSA demonstrated a markedly tortuous and dilated bilateral occipital artery and superficial temporal artery branch feeding into a scalp vein, and blood flow within the lesion is extremely fast (Fig. 2). The patient signed the consent form for the surgery and selected interventional embolization. The operation was performed under tracheal intubation and general anesthesia. After the 6F guiding catheter was placed into the bilateral external carotid artery, several branches of bilateral occipital artery and superficial temporal artery were selected, and then Onyx-18 liquid agent was used to embolize lesions. Again, angiography showed that the blood circulation of the posterior circulation involved in the lesion basically disappeared. Intraoperative blood loss was 5 ml. No severe surgical complications appeared after operation, and the patient was discharged 5 days later. A long-term clinical follow-up demonstrated the complete disappearance of pulsating mass in the region of the right top scalp, and cerebral angiography at 18 months showed no evidence of recurrence.

### Case 3 report

**Fig. 3:**
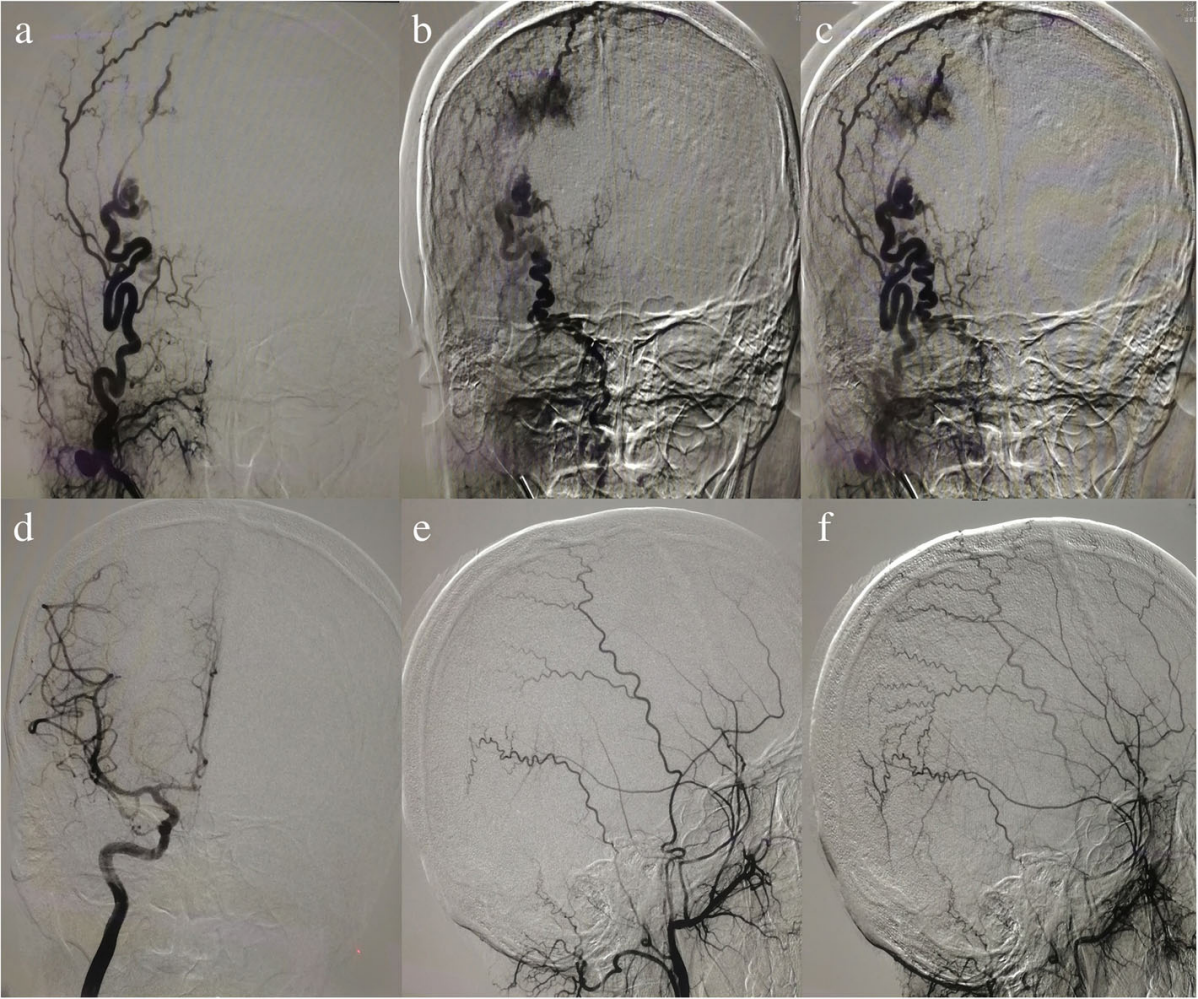
**a**–**c** Selective cerebral angiography demonstrated the presence of sAVF in the right occipital scalp region and the single right occipital artery supplied blood to the drainage of occipital scalp vein. **d**–**f** Angiographic examination at 6 months after surgery showed no signs of fistula

The other patient, a 26-year-old man, was admitted to our institution on October 14, 2017. The patient presented with pulsatile mass over the scalp without associated headache or dizziness. The patient had a history of brain trauma 8 years ago, and no family history of stroke or of other serious diseases. No history of surgery but a history of iohexol allergy. Physical examination indicated a 4 cm × 4 cm mass was located in the right occipital scalp and no other neurological positive signs. Post-admission CTA and further DSA confirmed the right occipital scalp arteriovenous fistula (right occipital artery supplied blood to the drainage of occipital scalp vein, Fig. 3), and the performance of cerebral angiography revealed no aneurysm or vessel abnormalities in other locations. After detailed information about the risk and the way of treatment, the patient chose surgical operation. The operation was performed under general anesthesia, and the patient takes the prone position. Scalp flap was devised depending on the scope of the lump and the cosmetic problem. Firstly, the right occipital artery was gently separated from the adjacent tissue, then it can be seen that the tension of the scalp mass decreased and the fluctuation disappeared after the blocking of the right occipital artery. Secondly, ablation electrode was used to coagulate the fistula, and finally, the diseased tissue was completely removed. Total excision of the scalp arteriovenous fistula was achieved, and intraoperative blood loss was less than 100 ml. Appropriate anti-inflammatory and analgesic treatment was given after operation. The patient recovered well, and no other complications developed. The pathologic examination strengthens the evidence of sAVF. The follow-up after 3 months and 6 months by physical and angiography examinations were normal without any signs of a fistula.

## Discussion

Scalp arteriovenous fistula (sAVF) is a rare vascular disease and previously named also as cirsoid aneurysms; therefore, only small case series have been reported by literature [1–6, 10]. It is widely believed that sAVF may be of either congenital or posteriori reason, and the latter one is the most common cause of lesion, which mainly results from head trauma or iatrogenic injury such as head surgery, hair transplantation, and scalp vein infusion [1–4, 7–10]. In most of the patients, superficial temporal artery (STA) was involved. After injury of the STA, hemodynamic begins to change and is characterized by high flow from the abundant feeding artery leading to a progressive venous dilation in the scalp [2, 3, 5, 8, 9]. The sAVF generally present with small subcutaneous swelling in the beginning, which can enlarge to become a pulsatile scalp mass over time and cause severe cosmetic deformity [2, 8, 9]. These lesions can also bring about various clinical signs and symptoms such as tinnitus, headache, hemorrhage, epilepsy, local allodynia, bruits, and scalp necrosis [3–5, 8, 9, 11–15]. The three patients we presented showed a pulsatile mass on the scalp without other severe symptoms. When the main feeding arteries are depressed, pulsatile sounds on auscultation will decrease and eventually disappear [8, 16]. Although physical examination is generally sufficient for diagnosis of sAVF, cerebral angiography is the gold standard diagnostic tool and can clearly show the fistulas, supplying artery and drainage vein [8, 11, 14, 16, 17].

In recent reports, therapeutic regimen varied depending on the type and location of the disease; however, it is also opted by physician preference. Two treatment options are currently available for scalp AVF: surgical excision and intravascular embolization. In the 27 cases recently reported, 11 were by surgical resection, 14 by intravascular embolization, and 2 by a combined treatment. The indication of the operation includes prevention of bleeding and relief of the cosmetic problem of the pulsatile mass and other symptoms such as local allodynia and tinnitus [18, 19]. The cases of intravascular embolization increased compared before as a result of the improvement of interventional technique, but radical surgical excision is still a necessary therapeutic method [2, 14, 18]. The advantages of surgical resection are lower costs, low incidence of complications, and lower recurrence rates, as well as it does not bear the risk of cosmetic deformity [4, 11, 18, 20]. Due to a high risk of recurrence in the arteriovenous fistulas, curative resection of these lesions after ligation of blood supply arteries is essential [11]. Scalp bleeding often occurred in those patients with incomplete excision of the fistula [14, 18]. Besides, primary closure of the scalp may not be easy after total excision of complex sAVFs, which is also prone to scalp necrosis. To avoid massive bleeding, detailed dissection of the abnormal vessels and careful removal of the lesion are recommended [14]. In order to achieve a completely cured surgical outcome, it is necessary to ligate the proximal feeder and remove the draining veins [4, 11]. Postoperative vasospasm is also a possible reason that may lead to a wrong thought of complete excision of sAVF [18]. The long-term follow-up of our patients showed no problems or recurrence. Therefore, cerebral angiography should be performed several months after operation to ensure the complete excision of lesion as well as no any recurrence.

Although total resection of the vascular malformation can achieve a perfect cure, ligation of proximal feeders may lead to distal tissue ischemia or insufficiency of the resection may bring about patency of the fistula due to collateral feeding artery [9]. In addition, complete resection may often lead to massive hemorrhage if the fistula is inadvertently punctured [1]. With the advances in interventional techniques and embolization materials, intravascular treatment is becoming more accepted as an efficient alternative to open surgery. The major point of endovascular therapy is to obstruct the abnormal arteriovenous shunt. Various intravascular approaches including transarterial, transvenous, and direct puncture for the remedy of sAVF have been reported in the recent literature [1, 5, 13, 16, 17]. The advantages of endovascular embolization are small trauma and quick recovery, but for many large supply arteries and draining veins, it is easy to recur after embolization, and the operation cost is high. At the junction of the feeding artery and the draining vein, the flow-guided microcatheter can be used to reach into the fistula. This is the main fistula location that must be successfully embolized to ensure full closure and also will not lead to ischemia in distal tissue due to interruption of arterial blood flow. Because of relative simplicity and safety, it is currently considered that the first choice of treatment is occlusion of fistula through Onyx liquid embolic material [3, 16, 17]. However, due to the extreme bending of the external carotid artery branches, transarterial access can sometimes be difficult to reach fistula. Especially in patients with complex multiple fistulas, it is difficult to fully obstruct all blood supply arteries by the transarterial approach [21]. So, interventional embolization for each blood supply artery may be inadequate or infeasible unless reaching into the venous side of fistula [1, 3, 5]. We reviewed the literature and found that two cases of recurrence were complex sAVF with multiple fistulas, in which surgical resection is also usually required and is facilitated by subtotal occlusion of this complex sAVF by embolization [17]. Besides, Cil B et al. reported successful treatment of sAVF by direct puncture [5]. In the this technique, usually under temporary compression of venous drainage, the sclerosing agent or embolic agent is retrogradely penetrated into the feeding arteries of the fistula and into the draining veins in the vicinity of the arteriovenous connection. The advantages of direct puncture are no use of microcatheters, less X-ray exposure, and short operation time.

## Conclusion

Either intravascular embolization or surgical resection is an effective therapy method for sAVF. Careful identification of angioarchitecture features contributes to successful treatment for the complex sAVF; therefore, it is recommended that neurosurgeons make the best treatment plan based on the location and number of the fistulas, feeding artery, and draining vein.

## Abbreviations

CT: Computed tomography
CTA: Computed tomography angiography
DSA: Digital subtraction angiography
ECA: External carotid artery
EJV: External jugular veins
FA: Facial artery
MMA: Middle meningeal artery
OA: Occipital artery
OV: Occipital vein
PAA: Posterior auricular artery
sAVF: Scalp arteriovenous fistula
SFV: Superficial frontotemporal vein
SOA: Supraorbital artery
SOV: Superior ophthalmic veins
SS: Sigmoid sinus
STA: Superficial temporal artery
Sv: Scalp veins
TS: Transverse sinuses
